# Single center experience on the rate of discontinuation and safety of cervical transforaminal epidural steroid injections using the modified approach in an office setting without sedation

**DOI:** 10.1016/j.inpm.2022.100075

**Published:** 2022-02-17

**Authors:** David Levi, Scott Horn, Dustin Runzo

**Affiliations:** a Jordan-Young Institute, Virginia beach, VA 23462, USA; b Eastern Virginia Medical School, Norfolk, VA 23507, USA

**Keywords:** Spine, Pain medicine, Transforaminal epidural steroid injection, Modified approach, Cervical, Radiculopathy, Safety, Radicular pain, Needle trajectory, Articular process, angle, Neck pain

## Abstract

**Background:**

Cervical transforaminal epidural steroid injections (CTFESI) are commonly used in the treatment of upper extremity radicular pain. Recently, a modification of the conventional technique has been developed and validated which has a theoretical safety advantage of less risk of needle contact of the spinal nerve and vertebral artery. The new approach involves a needle trajectory under fluoroscopic guidance which is directly based upon the specific superior articular process angle measurement on MRI.

**Objective:**

The purpose of this study was to evaluate the discontinuation rate of the modified approach CTFESI procedure in a non-sedated patient population. The study was also undertaken to confirm the safety of the procedure in an office-based setting.

**Methods:**

A retrospective review was performed of the authors' (DL, SH) practice to identify all CTFESI using the modified approach, performed between October 2018 through January 2021 through a query of the investigators’ (DL and SH) electronic medical record system. Any discontinued CTFESI procedure was identified. The reason for discontinuation was determined through medical record review. In addition, any significant neurologic or cardiovascular event occurring during or immediately following any completed or discontinued CTFESI was identified. Mild vasovagal reaction was not considered a significant complication.

**Results:**

A total of 973 CTFESI procedures were performed using the modified approach during the study period. Twelve procedures, 1.2% (95% CI 0.7–2.1%) were discontinued. Nine were aborted due to vascular flow not resolved with needle repositioning. Only three, 0.3% (95% CI 0.1–0.9) were aborted due to patient intolerance. There were zero significant neurologic or cardiovascular complications.

**Conclusion:**

The performance of the modified approach CTFESI appears to be well tolerated with a very low rate, 0.3%, of discontinuation due to intolerance in non-sedated patients. The zero incidence of neurologic or cardiovascular complication contributes to the current literature on the safety of this procedure.

## 1 Introduction

**Fig. 1:**
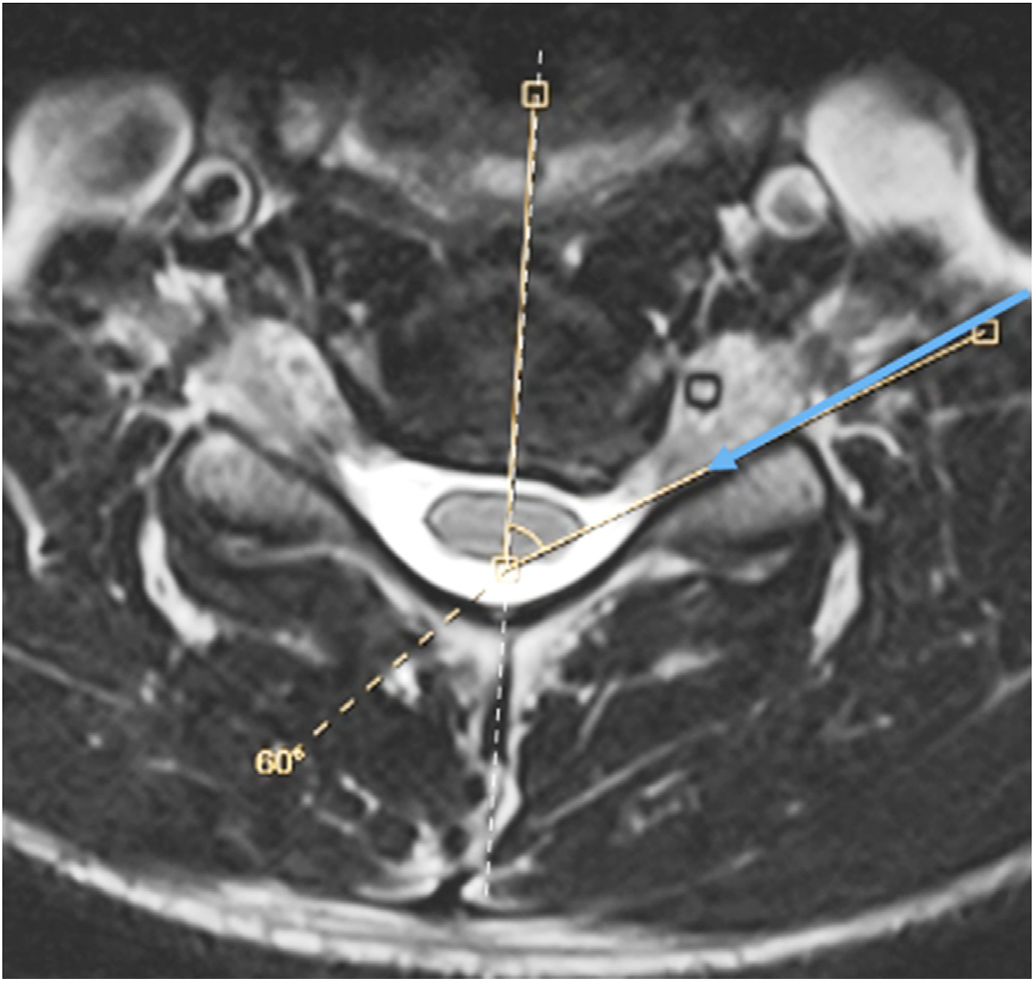
T2 Axial MRI of C5/6 with planned needle trajectory based upon measured angle of the ventral surface of the C6 superior articular process from the sagittal midline. As this SAP measured 60° on MRI. the fluoroscopic oblique angle would be preset at 60° relative to the fluoroscopic AP.

Cervical transforaminal epidural steroid injections (CTFESI) are commonly used in the treatment of radicular upper extremity pain [1]. The conventional technique is well described with needle trajectory based upon fluoroscopic oblique view in which the target foramen is “maximally tall, maximally wide” and “the superior articular process (SAP) is co-aligned with the laminae” [1,2]. Recently, a modification of the conventional technique has been developed which has a theoretical safety advantage of less risk of needle contact of the spinal nerve and vertebral artery [3,4]. The new approach involves a needle trajectory under fluoroscopic guidance which is directly based upon the specific superior articular process (SAP) angle measurement on MRI [3] Fig. 1. This approach was validated in a recent study demonstrating potentially superior contrast flow patterns compared to the conventional technique [3].

Although very rare, severe neurologic complications have occurred with CTFESIs [[5], [6], [7]]. While the modified CTFESI technique has been validated for contrast flow pattern, the approach has not been formally evaluated in a cohort for safety, beyond 100 patients [3]. The purpose of this study is to evaluate the safety of the modified approach to determine the incidence of severe complications. In addition, the study was undertaken to determine the rate and cause of discontinuation of the procedure in an office setting performed without intravenous sedation.

## 2 Methods

Formal IRB exemption was obtained from an independent IRB (Stirling IRB ID#: 8633-DLevi). A retrospective review was performed of the authors’ (DL, SH) practice to identify all CTFESI performed between October 2018 through January 2021 through a query of the electronic medical record system. Any severe neurologic or cardiovascular event, during the immediate or 24 ​h post-procedure period, was considered related to the procedure. Self-limited vasovagal reaction was not considered a significant complication. Surveillance for any severe complication outside of the immediate post procedure period was performed in the context of routine patient care. In addition, patients were routinely followed up at two weeks post procedure.

Any procedure with a discontinued modifier (modifier −53) on the electronic billing system was also recorded. A review of the medical record for these patients was performed to determine the cause for discontinuing the procedure.

## 3 Procedural technique

**Fig. 2:**
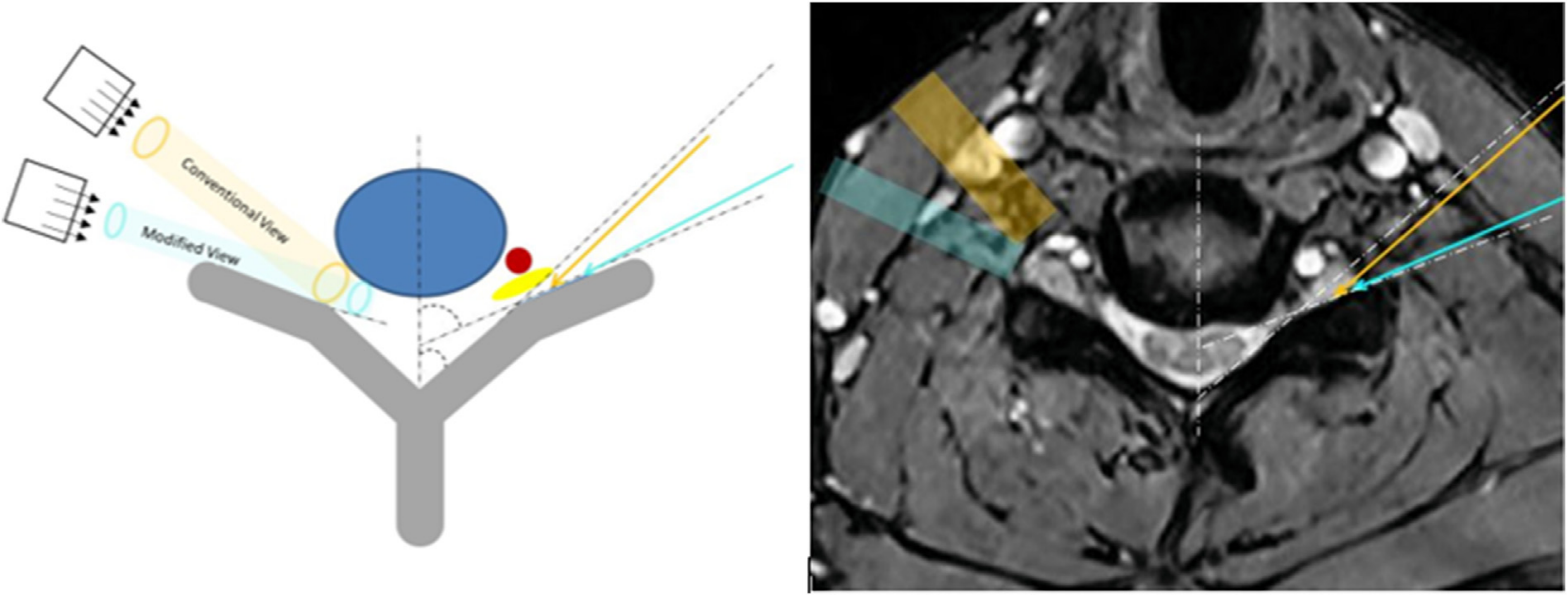
Cartoon with corresponding axial MRI demonstrating fluoroscopic view and needle trajectory of the conventional (orange) and modified (aqua blue) approaches. Note the proximity of the conventional needle to the spinal nerve.

**Fig. 3:**
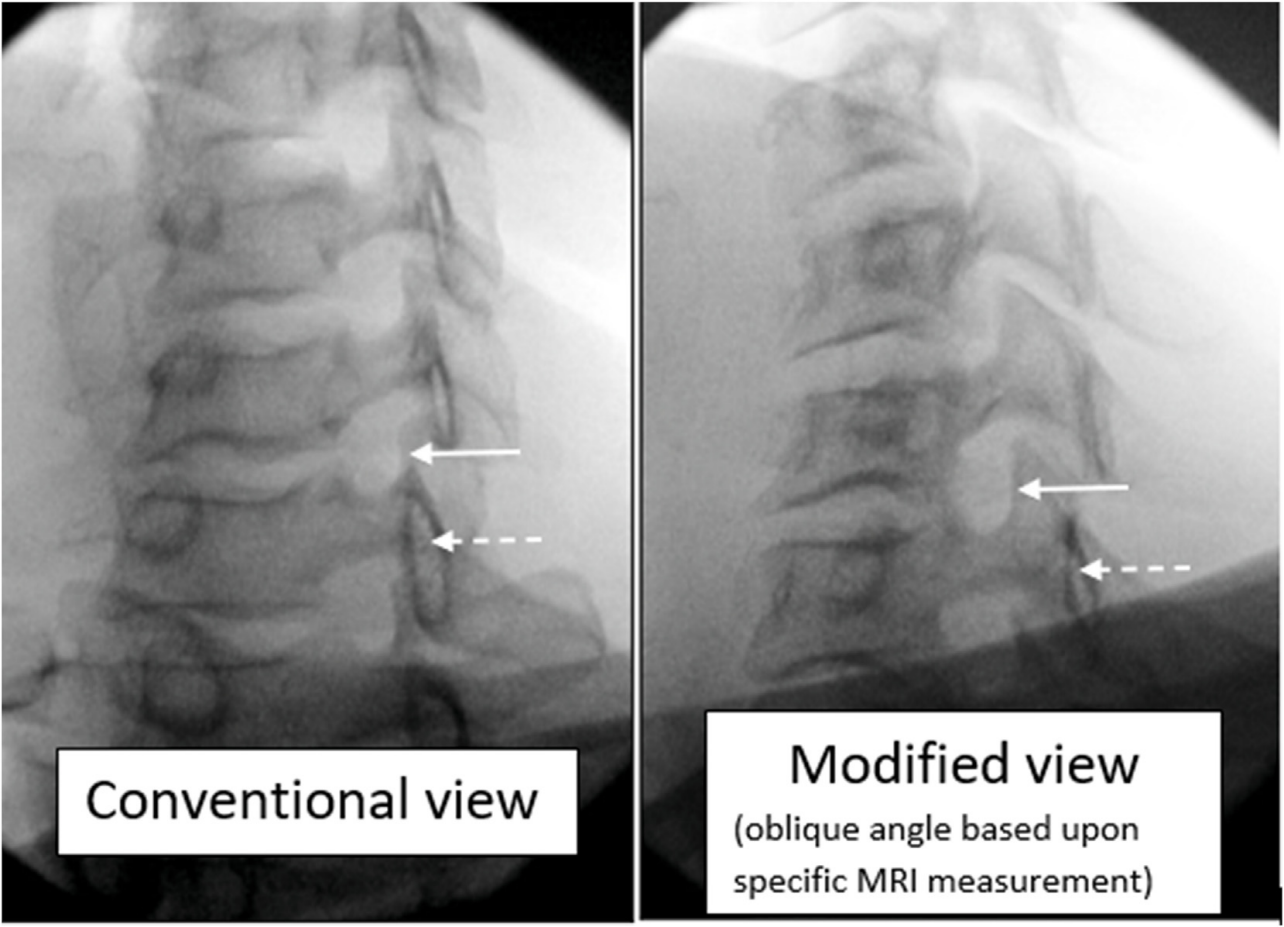
Fluoroscopic oblique images: Conventional view based upon co-alignment of the superior articular process (SAP) (solid arrow) and lamina (dashed arrow). Modified oblique view is based upon the obliquity of the specific angle measurement of the ventral surface of the SAP on MRI.

Performance of the modified CTFESI technique:

Informed verbal and written consent was obtained prior to all procedures. The approach was described in detail in a prior publication [3]. The technique requires the measurement of the superior articular process angle on the MRI (or CT) (Fig. 1), and presetting the fluoroscope at such angle for needle trajectory. The goal of the modified approach is to place the needle directly adjacent and parallel to the posterior wall of the foramen (the ventral surface of the SAP). This approach differs significantly from the conventional technique [1,3] (Fig. 2, Fig. 3).

**Fig. 4:**
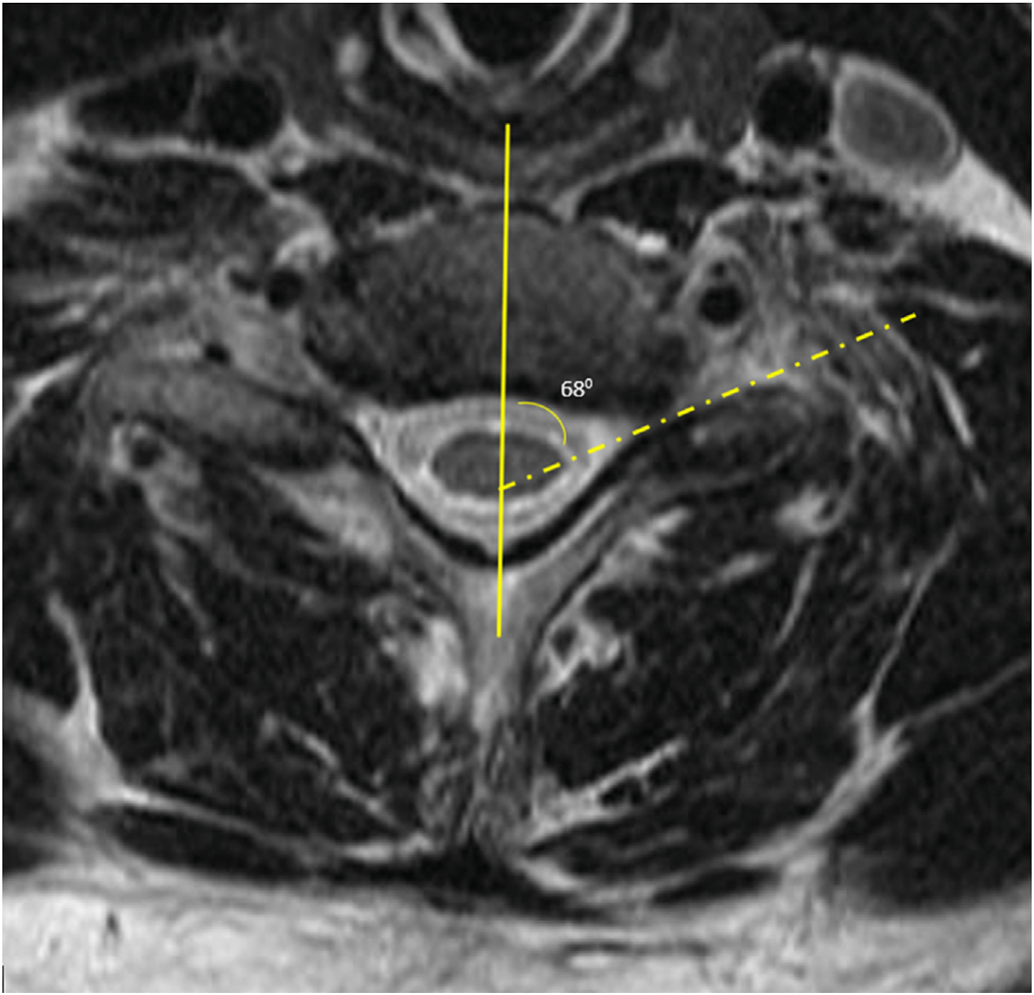
T2 Axial MRI measurement of the left C7 superior articular process ventral surface angle in preparation for a Left C6/7 TFESI. Initially a line is drawn in the sagittal midline bisecting the disc and spinous process (solid line). A second line is drawn along the ventral surface of the SAP (dashed line) to intersect with the sagittal line. The angle is measured (in this case, 68°) using the MRI angle measurement software.

**Fig. 5:**
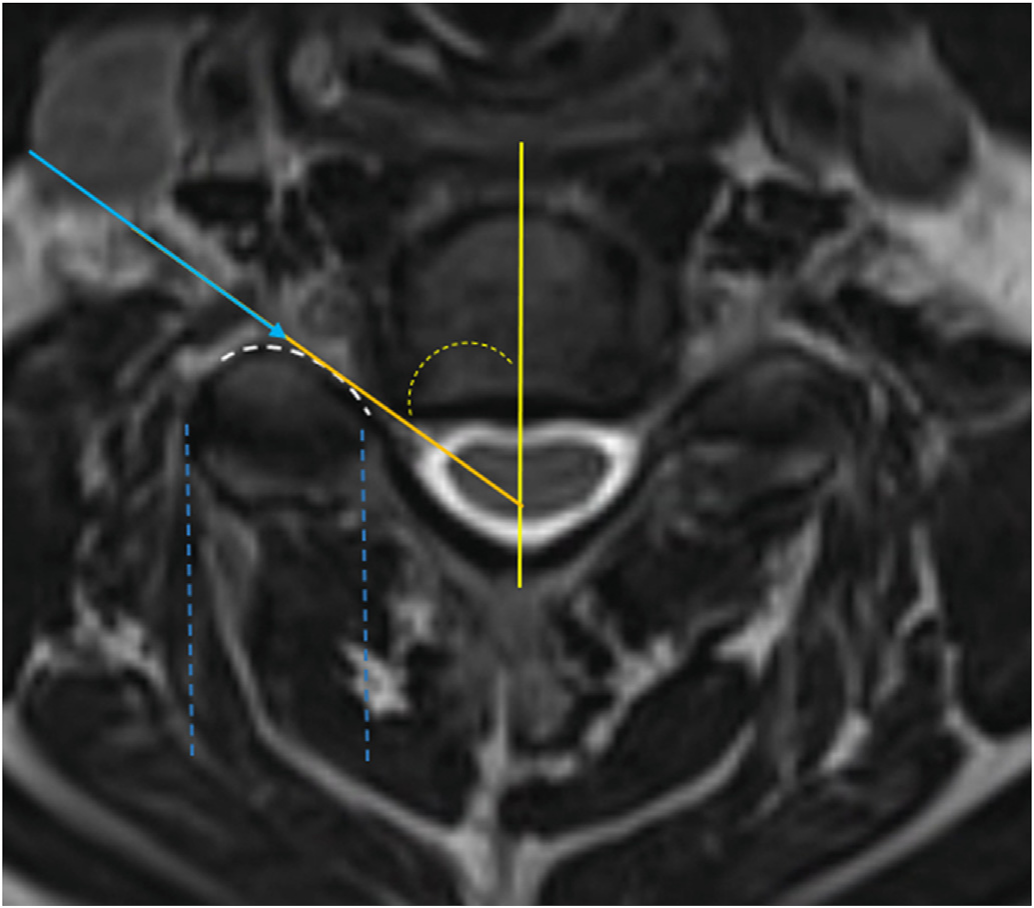
T2 axial MRI with curvilinear SAP morphology. Occasionally, the SAP is more of a curvilinear configuration rather than linear, as in Fig. 4. In such cases, the measurement procedure is similar to that demonstrated in Fig. 4 with a subtle modification; the line drawn along the ventral surface of the SAP (orange line) is tangential only to the medial half of the SAP which tends to be more linear. Note that a needle trajectory advanced at this given angle under fluoroscopy (solid blue arrow) is not likely to contact bone until the mid-portion of the articular pillar width (between dashed blue lines) when visualized on fluoroscopic AP view.

Pre-procedural MRI review to determine SAP angle measurement: Using the commonly available applications on MRI digital viewing platforms, lines and angle measurement tools are routinely accessible. Typically, T2 sagittal and axial series were chosen. Cross-section localization was performed to identify the index level and side for the planned injection. Usually, 2 or 3 axial slices are available for each foramen that include the inferior half of the SAP. Most often, the more inferior slice was chosen as it tends to have a more linear ventral surface of the SAP. Any of the slices along the inferior half of the SAP may have been selected for use. Based upon the cross-referenced sagittal series image, it was noted for future reference which part of the inferior half of the SAP was being measured as this would need to correspond to the needle positioning on the fluoroscopic procedure. The line drawing function was accessed in the MRI software tools. On the selected axial MRI slice, a sagittal line was drawn connecting the mid portion of the spinous process to the mid portion of the disc or vertebral body. This accounted for any rotation present on the MRI. Next a line was drawn obliquely along the ventral surface of the SAP intersecting with the sagittal line. The intersecting angle was measured using the angle measurement tool (Fig. 4). It was recognized that a small minority of SAP ventral surfaces are truly curvilinear. In such cases the medial portion of the SAP was typically linear and the oblique angle was usually based upon the ventral surface of the medial portion (Fig. 5). In each case, however, vulnerable structures such as the vertebral artery and the cervical or brachial plexus may be in this path of the needle trajectory. In these cases, the interventionalist would alter the approach angle to avoid these structures. This was of particular relevance when the medial portion of a curvilinear SAP was chosen for the needle trajectory, as this results in a more acute angle which places the vulnerable structures at higher risk.

**Fig. 6:**
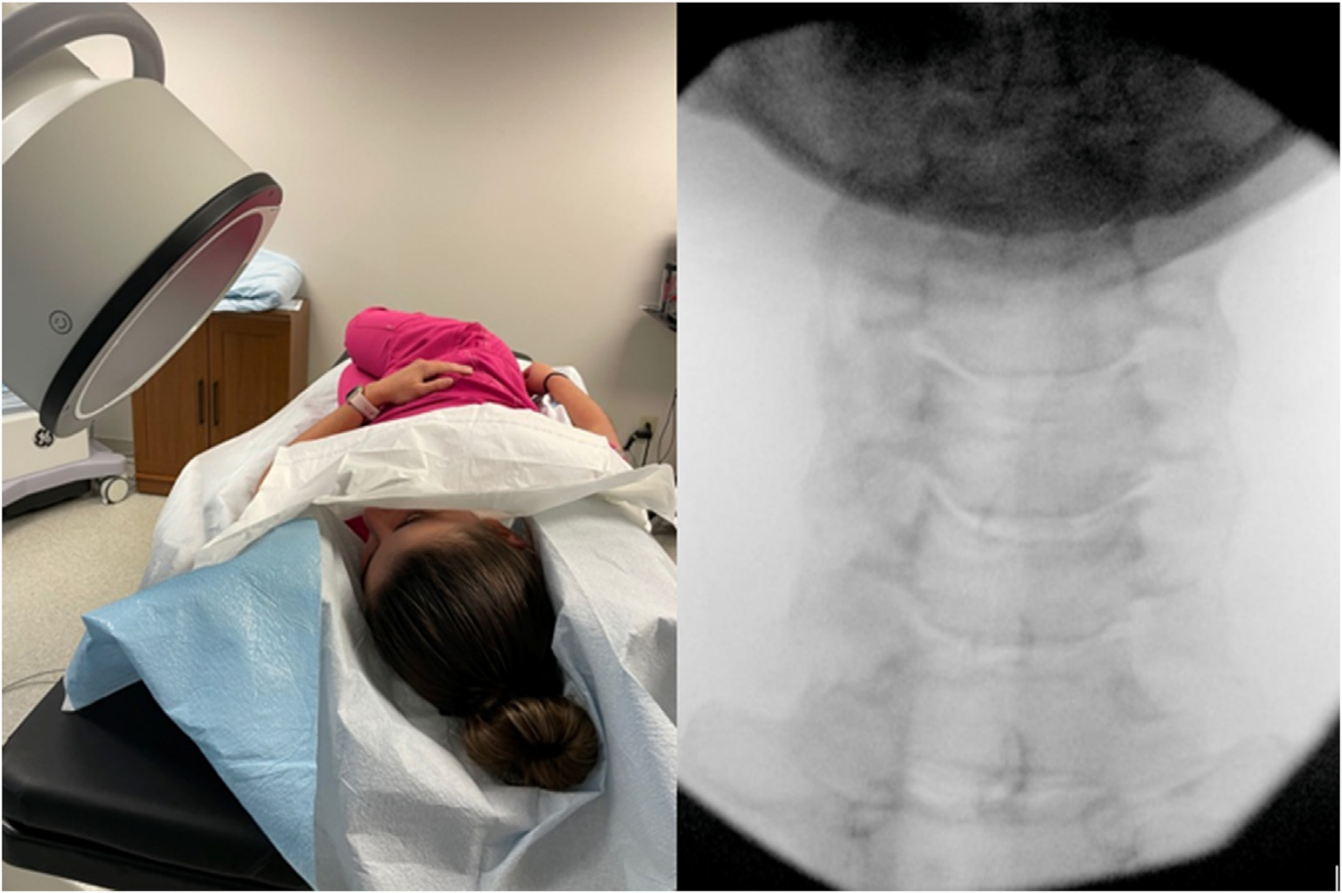
With the patient lying in an oblique position, ipsilateral side up, the fluoroscope is rotated to obtain a true AP fluoroscopic view. The angle of the fluoroscope is noted and used to compute the fluoroscopic angle to be used for needle advancement in Fig. 7.

**Fig. 7:**
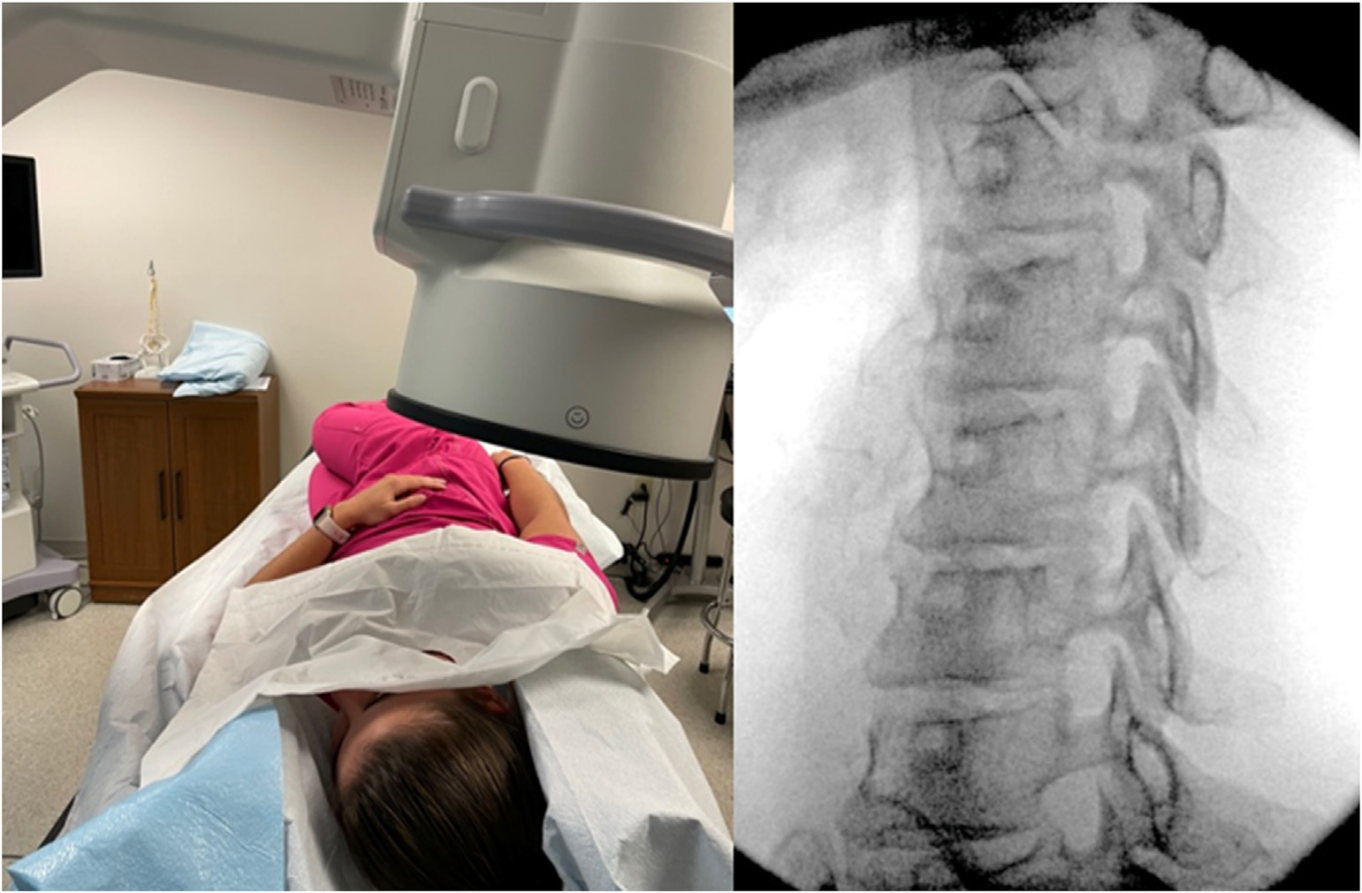
From the patients true AP position in Fig. 6, the C-arm is rotated ipsilaterally (past zero) to an angle based upon the relative angle of the patient's true AP as determined in Fig. 6. The true AP angle measurement (on fluoroscopy) is subtracted from the measured SAP angle on MRI. This follows the formula: Modified approach angle of obliquity for needle insertion ​= ​Angle of SAP measured on MRI minus the angle of the patient's true AP on fluoroscopy. In this case, the angle of the AP (Fig. 6) was determined to be 44°. The angle of the SAP on MRI was 61°. The C-arm was rotated ipsilaterally from 44°, past fluoroscopic zero degrees, to 17° (61–44 ​= ​17).

Determining the fluoroscopic angle based on the measured SAP angle on MRI**:** Typically, the patient was placed in an oblique position using foam wedges for both head and body support with the ipsilateral side up. The C-arm was then obliquely rotated to match the patient's unique oblique positioning. In this manner, the patient's true fluoroscopic AP view was obtained (Fig. 6). Typically, a caudal tilt of the image intensifier was also performed. Once the true AP fluoroscopic angle was established (matching the patient's oblique positioning), the fluoroscope was then rotated obliquely to the ipsilateral side to correspond to the measured angle on MRI. The degree to which the fluoroscope was ipsilaterally rotated to achieve the modified approach oblique view was based upon the following formula: Modified approach angle of obliquity ​= ​Angle of SAP measured on MRI minus the angle of the patient's true AP on fluoroscopy (Fig. 7). Caudal tilt of the image intensifier was further adjusted (typically 5–15°) to square the vertebral body endplates and bring the shadow of the contralateral pedicle into the superior aspect of the vertebral bodies of the target foramen.

For example, a patient is to undergo a right C5/6 TFESI. She is lying at an oblique angle with right side up. The C-arm image intensifier is rotated toward the patients left (contralaterally) to determine the patient's true AP based upon her oblique position, in this example, an angle of 44°. In this example, the patient's measured right C6 SAP angle on MRI is 61°. From the patient's true AP position of 44° to the patient's left (contralateral), the c-arm image intensifier would be rotated obliquely toward the patient's right (ipsilaterally) past 0° and then further ipsilaterally to 17°. (The true AP angle is subtracted from the measured angle on MRI to determine ipsilateral oblique angle.) If the patient were lying in a supine position, instead of at an oblique angle, and the true AP would still need to be determined under fluoroscopy. If the angle did happen to be zero degrees, the fluoroscope would be rotated directly ipsilaterally to the MRI SAP measured angle, in this case 61°. Most C-arms will be unable to accommodate larger obtuse angles for right sided injections. For this reason and ease of access bilaterally, the authors find the oblique patient positioning to be ideal.

**Fig. 8:**
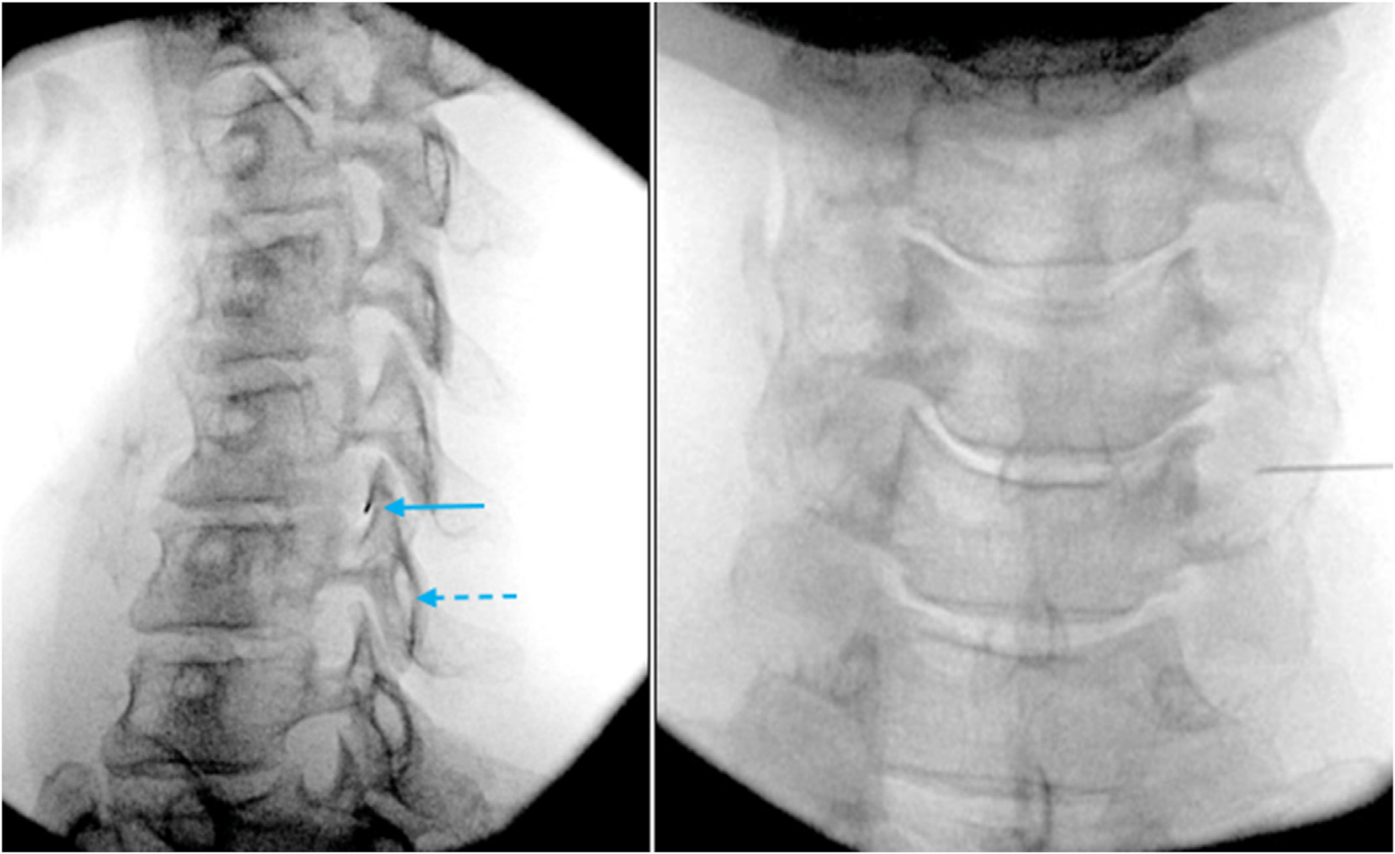
Oblique and AP fluoroscopic images of Right C6/7 TFESI with modified approach. The needle was advanced just ventral to the C6 SAP (solid blue arrow) using the specific oblique angle (as determined in Fig. 6, Fig. 7). In this oblique view the needle was advanced to a depth only to the lateral margin of the articular pillar. In the AP view the needle was further advanced to the approximate mid portion of the pillar. Note that there is no coalignment of the SAP (solid blue arrow) and lamina (dashed blue arrow) in this modified technique oblique view.

**Fig. 9:**
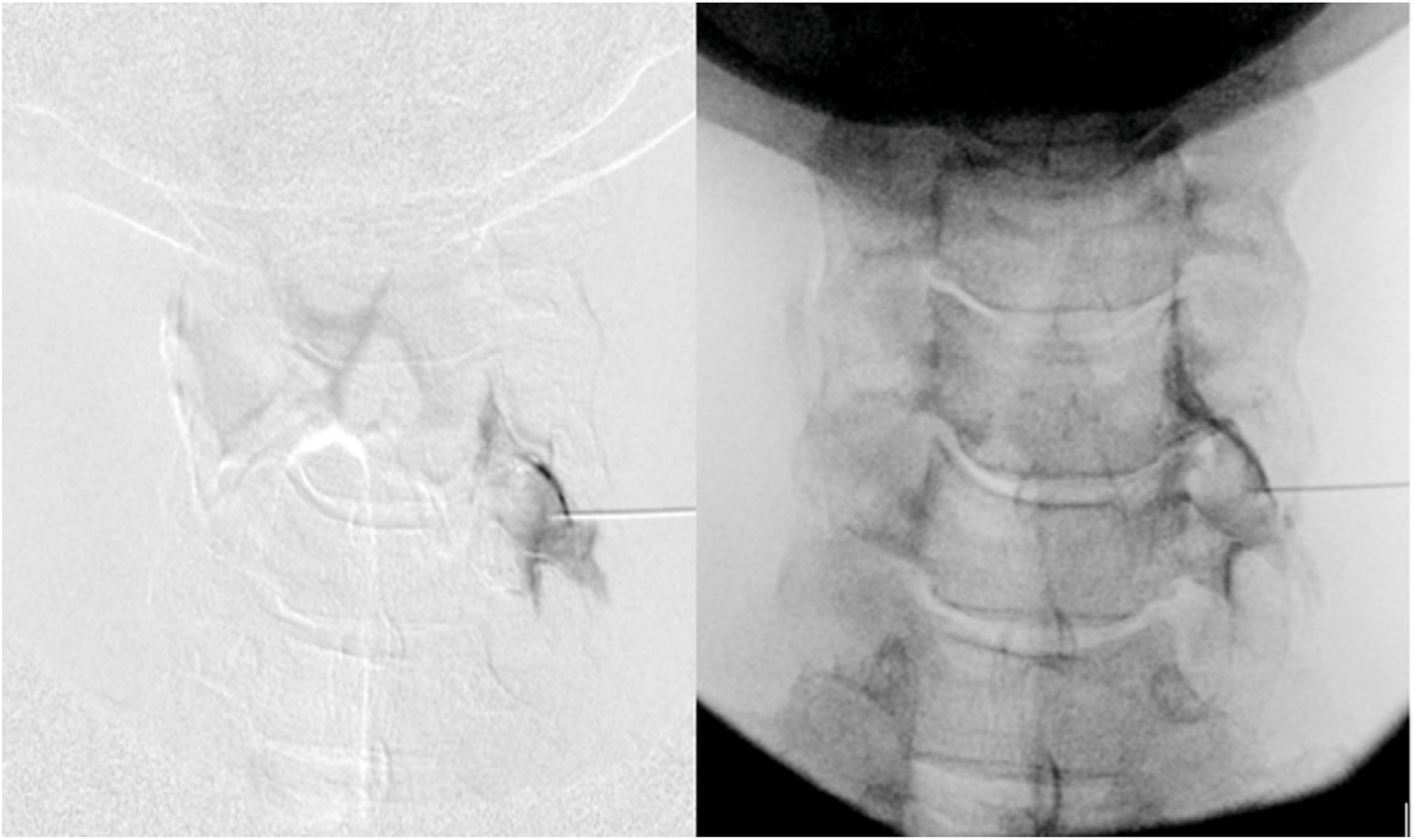
AP fluoroscopic images with digital subtraction imaging and conventional fluoroscopy demonstrating flow along the C6 spinal nerve and epidural flow.

Needle placement: Once the fluoroscope was set at the correct oblique angle, based upon MRI measurement, the ventral margin of the SAP was localized. Typically, a 2 inch 25-gauge needle was then placed at the skin entry site, just anterior to the ventral margin of the SAP. The needle was then advanced in line with the fluoroscopic beam with a very slight anterior to posterior trajectory (Fig. 8). The needle is typically advanced until there is contact on the SAP to determine the depth of the lateral edge of the foramen. The needle was then slightly redirected anteriorly to clear the lateral edge of the foramen and then was advanced 1–2 ​mm into the foramen. The needle at this point was then tangential to the SAP ventral wall. The fluoroscope was then rotated back to true AP to determine needle depth. While in the AP view, the needle was slowly advanced into the foramen, usually remaining in the lateral half of the articular pillar. Additional safety measures included multiple AP views during needle insertion, particularly if bone had not been contacted at an anticipated depth. In the AP view, under live fluoroscopy, using small volume extension tubing, 1–3 ​mL of contrast was injected. Digital subtraction imaging (DSI) was almost universally utilized (Fig. 9). If there was venous or inadequate flow noted on the injection of contrast, the needle was repositioned as necessary and further contrast was injected live with DSI. If arterial flow was encountered, the procedure was aborted. Once appropriate contrast flow was determined, a test dose of 1 ​mL of lidocaine was injected. This was then followed by 1 ​ml of dexamethasone 10 ​mg/ml. An additional 1 ​ml of contrast was then injected to increase the volume and clear the tubing.

## 4 Results

A total of 973 CTFESI procedures using the modified approach were performed on 697 patients. All procedures were performed by either DL (463; 47.5%) or SH (510; 52.4%). 97% were single level, unilateral procedures (Table 1).

**Table 1:** Patient demographics, number of levels and laterality.

	All procedures
Total	973 (697 patients)
Age of patients	Mean 58 years, range 24–92
Two level	28 (2.9%)
Laterality	Right 499 (51%) Left 474 (49%)

Of the 973 procedures, 12, 1.2% (95% CI 0.7%–2.1%) were discontinued on 11 patients. All terminated procedures were single level. Nine, 0.9% (95% CI 0.5%–1.7%) were aborted due to vascular flow not resolved with needle repositioning. Three, 0.3% (95% CI 0.1%–0.9%) were aborted due to patient intolerance. Five of the discontinued procedures were performed by DL, and 7 were performed by SH. Four were performed at spinal level C4/5, six at C5/6, and two at C6/7. No patients in the entire cohort received intravenous sedation. There were no severe neurologic or cardiovascular complications. (Wilson method used to determine 95% confidence interval) (Table 2).

**Table 2:** Discontinued procedures and complications.

Total discontinued procedures	1.2% (95% CI 0.7%–2.1%)
Patient age for discontinued procedure	Mean 67 years, range 48–86
Discontinued due to unresolved vascular flow	0.9% (95% CI 0.5%–1.7%)
Discontinued due to patient intolerance	0.3% (95% CI 0.1%–0.9%)
Significant neurologic/cardiovascular complication	0% (97.5% CI 0%–0.4%)

## 5 Discussion

The safety of CTFESIs has been a significant concern as case complications have been reported [7]. Unfortunately, many complications may not be published and therefore a true incidence in unknown [6]. A large multisite review of prospectively acquired data was published in 2016 to evaluate the safety of epidural steroid injections which included 1408 CTFESIs [8]. There were no severe complications reported in that cohort. Although the majority were performed using CT/fluoroscopy rather than just fluoroscopy as in the current study, this still may be applicable data as the modified approach used in this study has very similar needle trajectory and positioning to the CT/fluoroscopy guided technique [3,9]. Bush and colleagues reported the results of a prospective trial, including 1047 CTFESI procedures on 527 patients performed in an outpatient setting [10]. There were no severe complications. The Bush et al. study, however, may not be relevant to the safety profile being examined, as their needle positioning was not similar to the SIS guidelines approach [1] nor the modified technique [3].

The modified approach has an important potential safety advantage in avoiding critical vessel penetration. In a study of theoretical CTFESI needle trajectories in 312 MRIs, Karm et al. demonstrated zero incidence of vertebral artery penetration using the modified approach compared to 2–8%, depending upon the level, using an approach similar to the conventional technique [4]. The carotid artery also appears to be much less vulnerable to penetration with the modified approach, with a 0–1.6% incidence, compared to the conventional approach frequency of 2–19% [4]. Whether these theoretical safety advantages convey a clinical benefit is unknown and will require very large study populations.

The total discontinuation rate was found to be 1.2% (95% CI 0.7–2.1). Although the current study was not an analysis of patient tolerance of the procedure, we incurred a very low discontinuation rate, 0.3% (95% CI 0.1%–0.9%) due to patient discomfort. Vasovagal response was not responsible for any cases of discontinuation. Our findings were similar to that of El-Yahchouchi et al., reporting the exact same total discontinuation rate as this study of 1.2% for CTFESI, with vasovagal response accounting for only two patients [8]. No patients in this cohort received IV sedation for the procedure. It is well recognized that heavy sedation may increase the risk of complications during spinal injection procedures and should be avoided [11]. Rarely, patients in this study were pre-medicated with an oral anxiolytic for severe anxiety or needle phobia. Unfortunately, exact data of this small percentage was unavailable for this report.

**Fig. 10:**
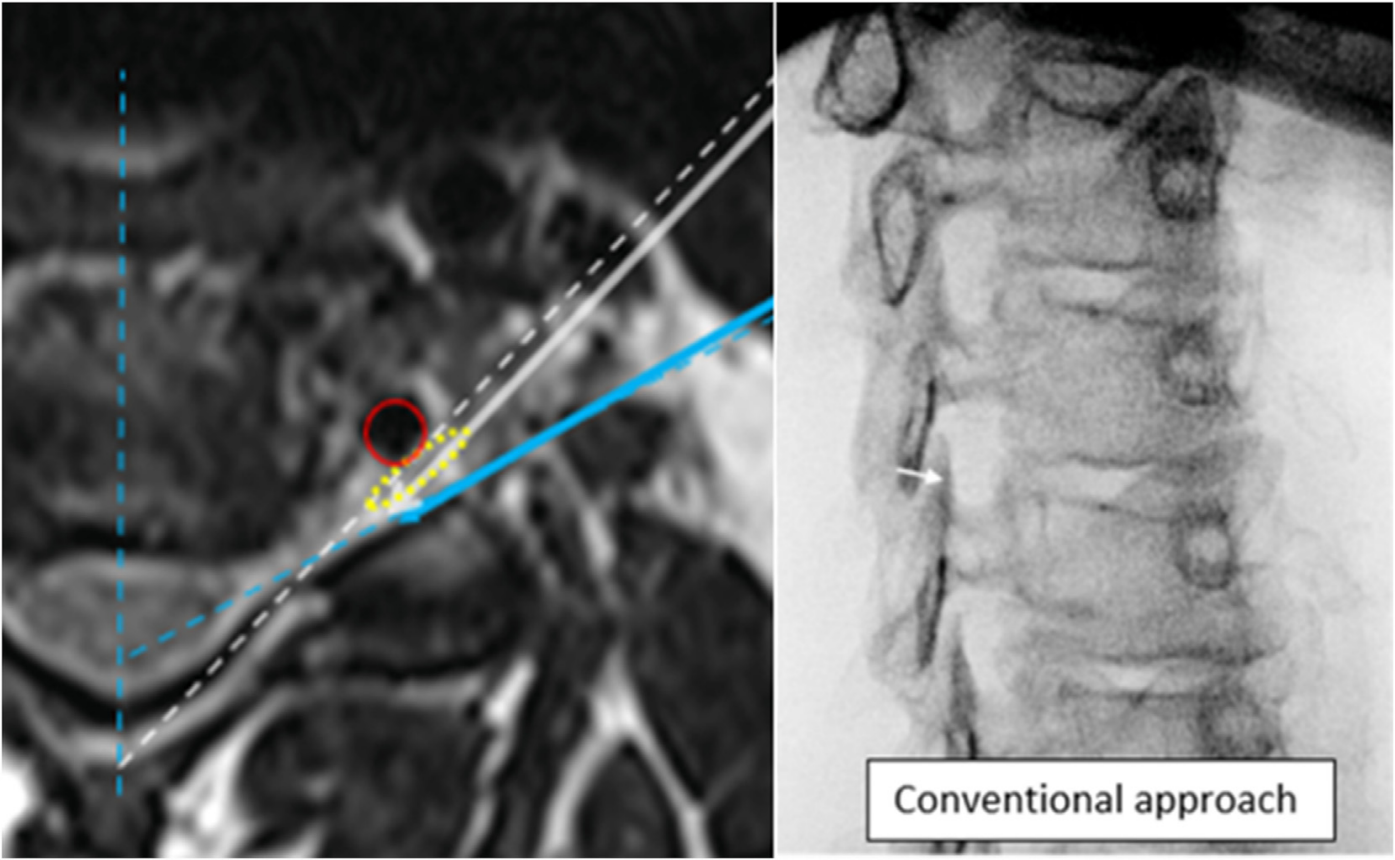
T2 axial MRI of a left C4/5 needle trajectory and corresponding conventional approach fluoroscopic oblique view demonstrating the higher risk of the conventional approach to contact the spinal nerve. MRI image demonstrates conventional approach (solid white arrow) compared to modified approach (solid blue arrow). To maximize the width of the visualized foramen on the fluoroscopic image, the oblique angle under fluoroscopy would be the same oblique angle as the lamina (dashed white line on MRI). Note that even with the conventional needle target point of one to two needle widths posterior to the visualized ventral portion of the SAP as indicated by the fluoroscopic image (arrow target point), the corresponding needle trajectory on the axial MRI contacts the neural tissue (yellow dotted oval). This contrasts with the modified approach needle trajectory (solid blue arrow) which remains clearly posterior to the neural structure. (For interpretation of the references to colour in this figure legend, the reader is referred to the Web version of this article.)

Although not specifically examined in this study, the modified approach may have a theoretical advantage for patient tolerance. When the needle is within the foramen, it lies within the vicinity of the spinal nerve. In either the conventional or modified approach, the needle tip should be posterior to this neural structure. The needle trajectory, however, in the modified approach may be less likely to contact the spinal nerve within the foramen compared to the conventional approach (Fig. 10). This is due to the modified technique trajectory which allows for greater tangential positioning to ventral surface of the SAP which directs the needle along a more posterior path. This more posterior trajectory also has a theoretical advantage of avoiding the cervical or brachial plexus. Contacting a nerve within the plexus may be responsible for the occasional radicular type pain encountered when the needle is advanced in the conventional approach and is still clearly superficial to the foramen.

The most common reason for procedure discontinuation was unresolved vascular flow. When venous flow occurred, the needle was slightly repositioned. If the venous flow did not resolve within two or three needle repositionings, the authors (DL and SH) would typically abort the procedure. If arterial rather than venous flow was seen, the procedure was terminated. Digital subtraction imaging was employed for nearly all CTFESI procedures with a few exceptions for severe pulmonary disease or movement disorders. Of note, one patient accounted for two discontinued procedures. This patient had persistent vascular flow during a right C4/5 TFESI which was reattempted 2 weeks later with the same result. The record was incomplete regarding the specifics of the type of vascular flow, venous or arterial. However, at least one of the nine procedures terminated secondary to vascular flow was due to arterial flow in an 81-year-old patient undergoing a left C6/7 TFESI.

There are several limitations of this report. The study was retrospective, and it is possible a severe neurologic or cardiovascular complication could have occurred without the authors’ knowledge although this is felt to be very unlikely. In addition to general surveillance of the patients during the first two days post procedure period, almost all patients were seen at two or three weeks for follow up. The follow up rate, although presumed to be close to 100%, is not available, and therefore, one cannot exclude the possibility that such an event went unreported. Another limitation of this study involves patient tolerance of the procedure. Although no vasovagal episode was responsible for procedure termination, the frequency of mild vasovagal symptoms occurring during and following the procedure was not available for this report and may be considered an important measure of patient tolerance.

## 6 Conclusion

This study demonstrates that the rate of discontinuation of CTFESI in an office setting is very low, 1.2%. The procedure appears to be very well tolerated in a non-sedated population. There were no significant neurologic or cardiovascular complications using a modification of the conventional CTFESI technique [3]. Although the number of procedures performed in this study, 973, is relatively small and is unlikely to detect severe complications considering the rarity, this report does serve to contribute to the overall denominator of published reports.

## Declaration of competing interest

The authors declare that they have no known competing financial interests or personal relationships that could have appeared to influence the work reported in this paper.
